# 

**Published:** 1849-07

**Authors:** 


☞ Several notices of new works, and other editorial articles are postponed, for want of space, until our next number.

